# Genetic analysis confirms the freshwater origin of the endemic Caspian sponges (Demospongiae, Spongillida, Metschnikowiidae)

**DOI:** 10.3897/zookeys.915.47460

**Published:** 2020-02-24

**Authors:** Agniya M. Sokolova, Dmitry M. Palatov, Valeria B. Itskovich

**Affiliations:** 1 N. K. Koltzov Institute of Developmental Biology, Russian Academy of Science, Vavilova str., 26, Moscow, 119334, Russia A. N. Severtsov Institute of Ecology and Evolution, Russian Academy of Science Moscow Russia; 2 A. N. Severtsov Institute of Ecology and Evolution, Russian Academy of Science, Leninskij prosp. 33, Moscow, 119071, Russia N. K. Koltzov Institute of Developmental Biology, Russian Academy of Science Moscow Russia; 3 Biological faculty, Moscow State University, Leninskie Gory 1 / 12, Moscow, 119991, Russia Moscow State University Moscow Russia; 4 Limnological Institute of Siberian Branch of Russian Academy of Science, Ulan-Batorskaya, 3, Irkutsk, 664033, Russia Limnological Institute of Siberian Branch of Russian Academy of Science Irkutsk Russia

**Keywords:** Caspian Sea, CO1, ITS1, ITS2, *
Metschnikowia
*, Porifera, Spongillida

## Abstract

The Caspian Sea is a unique inland brackish waterbody inhabited by highly endemic fauna. This fauna consists of species of both marine and freshwater origin. Some Caspian invertebrates cannot be confidently referred to as animals of either origin. The endemic monophyletic family of sponges, Metschnikowiidae, is among them. Although these sponges are considered as fresh water in the modern literature, no researcher has seen them alive for many years, and its status is actually unconfirmed. Here, we present the first photos of *Metschnikowia tuberculata* Grimm, 1877 and report evidence for its freshwater origin based on analysis of ITS1 and ITS2 sequences and partial sequences of CO1 gene. According to the genetic analysis, *M. tuberculata* belongs to the order Spongillida. We observed specimens of diverse appearance, but their spicule complement proved to be similar, and ITS sequences were identical. Thus, we conclude that they belong to the same species. The obtained results expand our knowledge about the dispersal ability of freshwater sponges.

## Introduction

The Caspian Sea is the largest enclosed inland waterbody on our planet, variously classed as the world’s largest lake or a full-fledged sea. Being the residue of ancient seas, the Caspian Sea is completely isolated from oceans now. It is a nondrainage brackish waterbody with profound seasonal and multiannual level oscillations (Kostianoy and Kosarev 2005; Chen et al. 2017). Because of several transgressions and regressions, the history of the Caspian biota is dynamic: periods of faunal isolations and species extinctions alternated with species invasions (Grigorovich et al. 2003). The present fauna comprises nearly 46% of Caspian endemics and 20% endemics of the Ponto–Caspian region (Zenkevich 1963). In recent years, the invasion of a few species decreased the endemism percentage, but it is obviously still significant. The Caspian endemics fall into four groups: (1) species of Tethyan origin (e.g., sturgeons, gobiids and clupeids); (2) species originating from brackish Sarmatian or Pontic Lakes, (e.g., the onychopod Cladocera); (3) opportunistic freshwater species (most Rotifera, the non-onychopod Cladocera, most cyprinid fish); (4) a few invaders of northern (Baltic and White Seas) origin, that show few signs of speciation (copepods *Limnocalanus grimaldii* (Guerne, 1886), several mysids, the Caspian salmon, the Caspian seal) (Dumont 1998). These endemic animals together with freshwater species and species of Atlantic–Mediterranean and Arctic origin represent the current biodiversity of the Caspian Sea (Grigorovich et al. 2003).

In the last century, the Caspian Sea was actively studied, but after the disintegration of the USSR, most of the research ceased. However, some of the Caspian species did not get due consideration even in favorable times. This particularly applies to sponges (phylum Porifera). The endemic Caspian sponges were first described by Grimm in the 19^th^ century (Grimm 1876, 1877). He reported four species: *Reniera flava* Grimm, 1876, *Amorphina caspia* Grimm, 1877, *Metschnikowia tuberculata* Grimm, 1877 and *M. intermedia* Grimm, 1877. Later, Chernjavsky (1880) described a fifth species, *Amorphina protochalina* Czerniavsky, 1880, based on a collection of Ulskiy (expedition of Ivashincev, 1856–1867). Then Dybowsky (1880) proposed to synonymize *R. flava* and *Metschnikowia flava* and detailed descriptions of the three *Metschnikowia* species based on, apparently, Grimm’s material. After more than 80 years, Koltun (1962) reviewed Grimm’s collection and joined *R. flava*, *M. tuberculata* and *M. intermedia* into the only one species, *Metschnikowia tuberculata*, although he did not find the original holotypes. As for *Amorphina* species, Koltun succeeded in finding the holotype of *A. protochalina* and suggested it to be an aberrant form of *M. tuberculata*. The only found fragment of *A. caspia* was regarded by him as not fitting the original description. Thus, the two *Amorphina* species were claimed as doubtful and requiring confirmation. In a later comprehensive taxonomic revision of sponges, the Caspian sponges became considered as an endemic family with only one species *Metschnikowia tuberculata* (Manconi and Pronzato 2002).

The authors concerned with *Metschnikowia* surmised its relation to marine sponges: Grimm (1877) supposed it to be close to Renieridae or Suberitidae, Lundbeck (1902) assigned it to Renierinae, Annandale (1914) suggested its relationship with *Reniera*. The habitus and spicule shape (acanthoxeas) of *Metschnikowia* convinced Koltun (1962), after Martinson (1940), to regard it as a transitional form between marine and freshwater sponges. Manconi and Pronzato (2002) placed the family Metschnikowiidae within the freshwater sponges, suborder Spongillina (now order Spongillida). Morrow and Cárdenas (2015) proposed metschnikowiids to be reallocated to marine Haplosclerida, based on their brackish habitat and morphological affinities with *Janulum*, a marine haplosclerid (although *Janulum*’s skeleton comprises acanthostrongyles but not acanthoxeas).

Thus, given the fact that the Caspian fauna includes species of both freshwater and marine origin, the evolutionary history of sponges remained unknown. So did their actual diversity and ecology because it has been a long time since biologists saw them alive. The current study presents the first photos of live Caspian sponges and their spicules, and the genetic analysis revealing their phylogenetic position.

## Material and methods

### Sampling procedures

The material was collected in the vicinity of Aktau town, Kazakhstan (44°04'88"N, 50°86'98"E), in September 2018. Specimens were gathered by SCUBA diving, snorkeling or by turning over littoral stones. Sponges were carefully detached from rocks and lower parts of large stones by forceps or were collected with the substratum (*Mytilus* aggregations). Specimens were fixed in 96% ethanol and RNA-later. When possible, sponges were photographed *in situ* before collection. For comparative purposes, specimens of *M. tuberculata* from museum collections (deposited in the Zoological Institute of the Russian Academy of Science (ZISP) and partly in the Zoological Museum of Moscow University (ZMMU)) were also investigated.

### Morphological analyses

For scanning electron microscopy (SEM), spicules were purified with potassium dichromate solution and mounted on a stub according to the classical method (Manconi and Pronzato 2000). Measurements of length and width of spicules were taken with light microscopy.

### Genetic analysis

Total genomic DNA extraction was performed using the RIBO-sorb RNA/DNA extraction kit (InterLabService, Russia). The 676 bp fragment at the 5’ end of the CO1 gene was amplified and sequenced using universal barcoding primers (Folmer et al. 1994). ITSs were used as a main marker with the best resolution since many species of freshwater sponges have the identical COI sequences (Erpenbeck et al. 2011; Itskovich et al. 2013; Carballo et al. 2018). Previously described primers (Itskovich et al. 2017) were used for amplification of ITS1, 5.8S rDNA and ITS2. Polymerase chain reaction (PCR) amplifications of ITS1 and ITS2 were performed on a DNA Engine Dyad thermal cycler (Bio-Rad, USA) using the 5*ScreenMix (Evrogen). The cycle parameters were initial denaturation at 94 °C for 120 s, followed by 35 cycles of denaturation at 94 °C for 30 s, annealing at 55 °C for 30 s and extension at 72 °C for 120 s, followed by a final extension of 8 min at 72 °C. Each PCR product was purified by electrophoresis in 0.8% agarose gels and eluted by freezing and thawing. Sequencing of both strands of each PCR product was carried out by Syntol (Russia) using a BIG DYE 3.1 terminator mix on an ABI 377 Sequencer. Chromatograms were analyzed using BioEdit 5.09 (Hall 1999). All sequences have been deposited with GenBank (http://www.ncbi.nlm.nih.gov) with the accession numbers MK659927–MK659935 (ITS1 and ITS2) and MN431221–MN431229 (CO1). The assignment of the sequences obtained from Porifera was performed using the BLAST software program (http://www.ncbi.nlm.nih.gov/blast/). Sequences were initially aligned using ClustalW 1.7 (Thompson et al. 1994) under default parameters including all available sequences of ITS1 and ITS2 of freshwater sponges available from GenBank, with mandatory manual correction. Phylogenetic trees were constructed using the maximum likelihood (ML) method and Bayesian inference (BI), as implemented in MEGA 5 (Tamura et al. 2011) and MrBayes 3.1.2 (Ronquist and Huelsenbeck 2003). Genetic distances in pairwise comparisons between all analyzed sequences were calculated according to Kimura’s 2-parameter model. For the ML analysis, the HKY+G (CO1) and K2P+G (ITS1 and ITS2) models were best fitting. The robustness of the ML trees was estimated by bootstrap percentages (Felsenstein 1985) using 500 replicates with heuristic search and stepwise addition starting trees.

Bayesian analyses on nucleotide sequences were run with a parallel version of MrBayes 3.1.2 (Ronquist and Huelsenbeck 2003). Each Bayesian analysis comprised at least two simultaneous runs of eight Metropolis-coupled Markov chains at the default temperature (0.2 °C) under the most general model (GTR+G+I) because overparameterization does not negatively affect Bayesian analyses (Huelsenbeck and Rannala 2004). Analyses were terminated after the chains converged significantly, indicated by the average standard deviation of split frequencies <0.01. The robustness of the Bayesian trees was estimated by posterior probabilities.

*Trochospongilla latouchiana* Annandale, 1907 (Spongillidae) was used as the outgroup for ITS sequences because early branching of this genus among Spongillida has been shown in previous phylogenetic reconstructions (Addis and Peterson 2005; Itskovich et al. 2008). This is the most distant taxon of sponges whose ITS sequences are able to be aligned with the sequences of metschnikowiids. *Vetulina stalactites* Schmidt, 1879 was used as the outgroup for the CO1 sequences as closest marine group (Schuster et al. 2018a).

The World Porifera Database (Van Soest et al. 2020) was used for checking statuses of the taxa under discussion.

## Results

Sponges were abundant in the studied depths (0.5–5 m) but preferred hidden places. A total of 41 sponges were collected; nine of them were sequenced.

### General morphology

**Figure 1.**
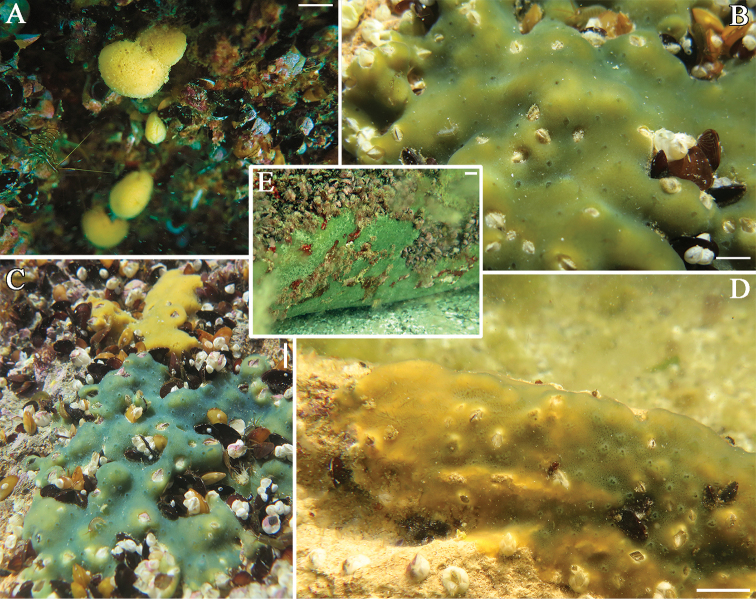
In situ photos of *Metschnikowia tuberculata*. **A** Globular morph **B–E** encrusting morphs. Scale bars: 2 cm (**A, E**), 1 cm (**B, C, D**).

We observed sponges of highly variable appearance: crusts of yellow, blue, green or several/ transitional colors and bright-yellow spheres (ø 2–7 cm) (Fig. 1). Worth noting, the sponges of spherical shape were always of yellow color, while the encrusting shape was not associated with a certain color. Oscula were clearly noticeable, quite regularly arranged, sometimes slightly raised above the surface. In some cases, exhalant canals of star-like structure were seen (Fig. 1 D). One of the encrusting sponge morphs differed from others by having smaller oscula, large body area (up to 70 cm^2^), reduced thickness (3–4 mm) and distinctive faded-green color (Fig. 1 E).

Thus, two stable morphs could be distinguished among others: thin faded-green encrusting sponges (Fig. 1E) and spherical bright-yellow sponges (Fig. 1A).

### Habitat

The yellow globular morph and faded-green encrusting morph occupied exclusively shaded areas of rocks and lower surfaces of large stones at a depth of 2 m and more. Encrusting sponges of other colors were observed on less-shaded substrata as well as on shaded surfaces at a depth of 50 cm and more.

### Spicule characters

**Figure 2.**
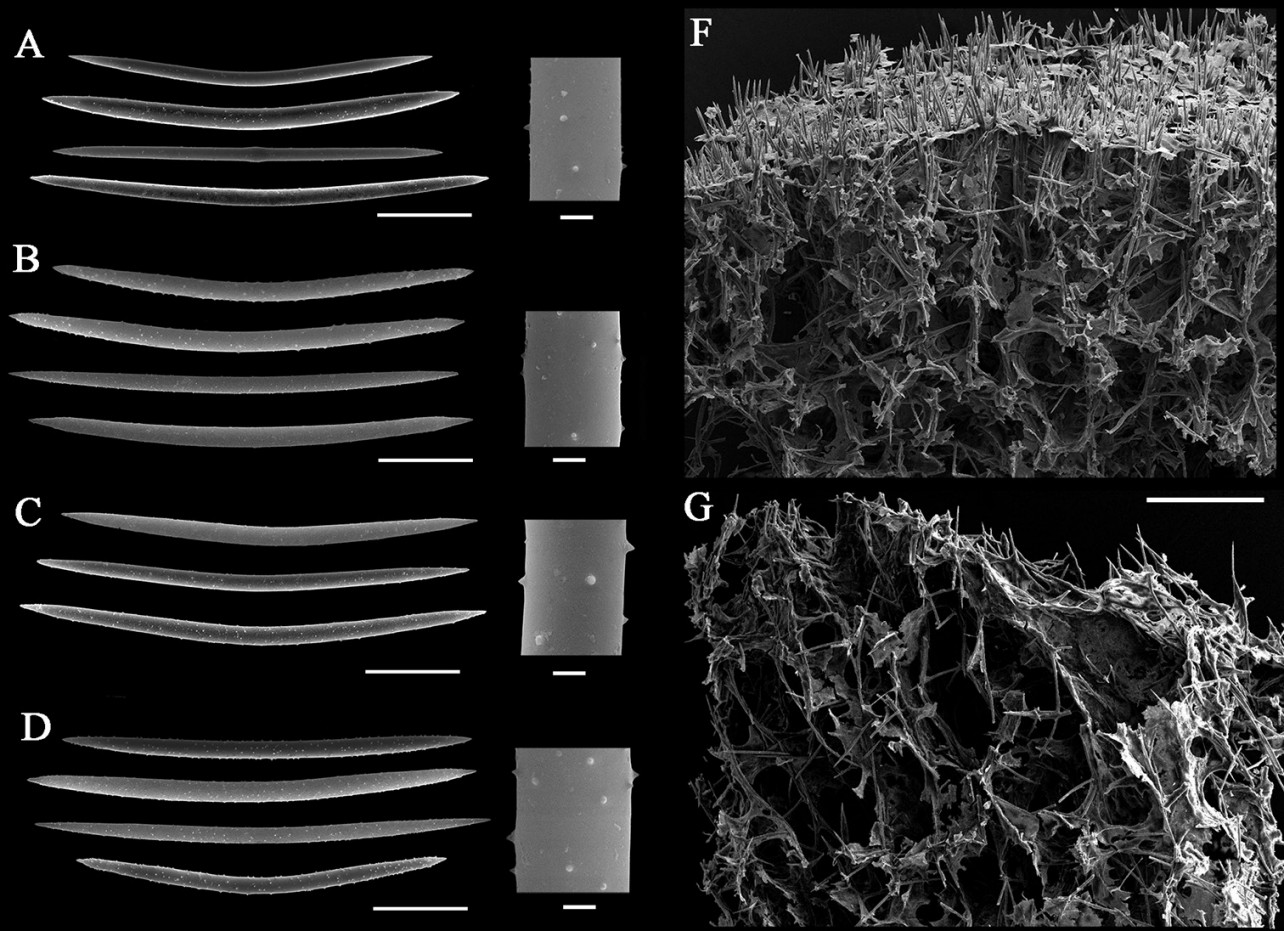
Skeleton complement of *Metschnikowia tuberculata*. **A–C** Spicules of encrusting sponges **D** spicules of a globular sponge **E** skeleton arrangement of a globular sponge **F** skeleton arrangement of an encrusting sponge. Scale bars: 30 µm (**A–D** whole spicules); 2 µm (**A–D** magnified parts); 250 µm (**E, F**).

Spicules were 126–175 µm × 3–7.5 µm (for more details see Table 1), slightly curved or almost straight oxeas, from finely spiny to greatly spiny (Fig. 2). Sponges of different shapes and colors have no significant distinctions in spicule characters, although oxeas of the yellow globular morphs are generally spinier (Fig. 2D) than in encrusting sponges (Fig. 2A–C). Size and density of spicule spines vary within a specimen.

**Table 1.** Measurements of spicules of *Metschnikowia tuberculata*. YGC – yellow-green crust, YS – yellow sphere, FGC – faded-green crust, BC – blue crust; SD – standard deviation.

Specimen	Appearance	Spicule length (*N* = 25)				Spicule width (*N* = 25)			
		min	mean	SD	max	min	mean	SD	max
Present collection									
	YGC	126.25	**145.33**	7.69	157.50	3.75	**5.50**	0.91	7.00
	FGC	125.00	**152.40**	10.69	175.00	4.00	**5.68**	0.82	7.50
	BC	141.25	**152.50**	5.33	160.00	4.75	**5.82**	0.82	7.25
	YS	140.00	**155.14**	6.73	163.75	3.00	**5.03**	0.72	6.50
	YS	132.50	**144.20**	6.57	155.00	3.75	**5.60**	0.92	7.25
Museum collection									
M. t. var. tuberculataZMMU, specimen №252	Unknown	143.00	**148.42**	4.46	156.00	4.00	**6.50**	0.99	7.90
M. t. var. tuberculataZISP, slide collection, slide №10500	Presumably YS (Fig. 3A, upper)	157.50	**173.26**	7.91	183.25	10.25	**14.00**	1.38	16.25
M. t. var. intermediaZISP, slide collection, slide №10473	Unknown	117.75	**148.16**	11.81	165.00	4.75	**5.90**	0.81	7.50
M. t. var. flavaZISP, slide collection, slide №10529	Unknown	151.25	**172.15**	10.59	192.50	7.50	**12.94**	1.43	15.00
Museum collection, measurements from Koltun (1962), based on numerous specimens									
M. t. var. tuberculata		130			190	12			18
M. t. var. intermedia		120			210	8			19
M. t. var. flava		120			200	10			17

### Skeleton arrangement

Oxeas constitute paucispicular ascending tracts, sometimes organized in quite regular anisotropic reticulation. The degree of regularity highly varies, and it is more prominent in peripheral parts of sponges. In encrusting forms, tracts protrude outward from the sponge surface (Fig. 2F). In globular sponges we observed a smoother surface with only single spicules piercing the surface layer (Fig. 2E). Spongin is sparse; the basal spongin plate was not found in our material.

### Comparison with museum collection

**Figure 3.**
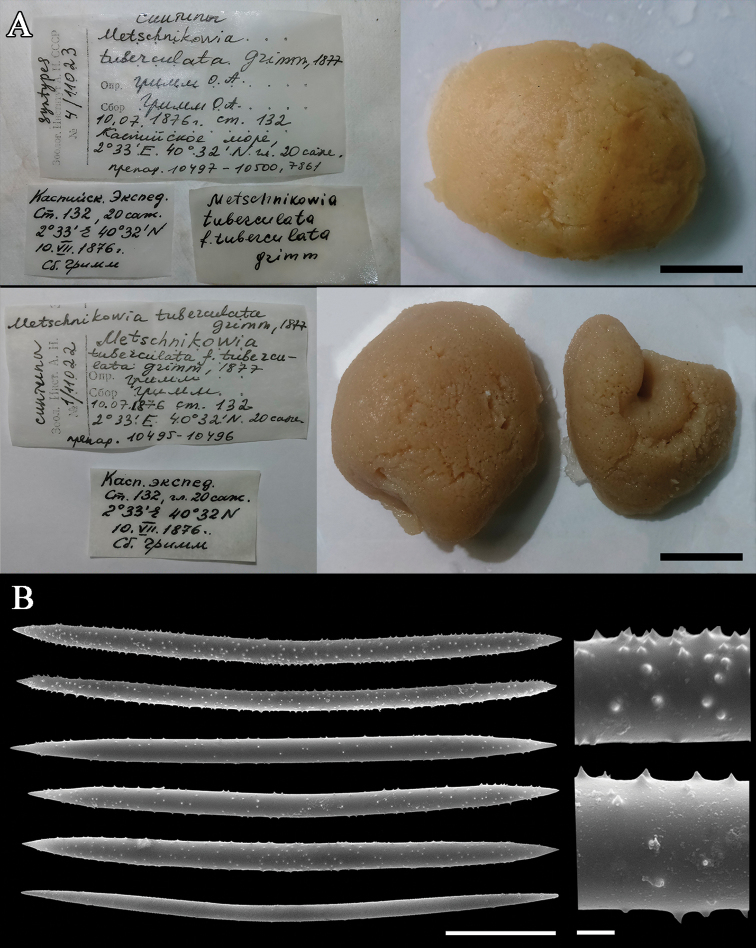
Museum specimens of *Metschnikowia tuberculata*. **A** General view of the three syntypes and their labels. Scale bar 1 cm. ZISP, specimen №11023. Collector: Grimm O. A. 10.07.1876, station 132; coordinates 2°33'E, 40°32'N, depth 20 fathoms (42.6 m). Associated slides: 10497–10500 (upper specimen) and 10495–10496 (lower specimens) **B** spicular complement of M. tuberculata var. intermediaZMMU, specimen №251. Scale bars 30 µm (whole spicules) and 2 µm (magnified part). *The longitude is counted from Baku, i.e., 2°33'E = 52°23'E

The only existing museum collection of the Caspian sponges includes spirit specimens (ZISP № 10994–11030, ZMMU № 251–252) and slide preparations. This collection was mainly composed of sponges gathered by Grimm in the 19^th^ century, but now the majority of his material is, apparently, lost. There are also some sporadic specimens from later expeditions. The wholly preserved sponges are often of spherical shape and look identical to the globular yellow sponges collected by us (compare Fig. 1A and Fig. 3A). Their spicule composition has no general differences with spicules of sponges from the Aktau vicinity, but their oxeas have larger spines distributed more densely (Fig. 3B). Spicule size of the museum sponges varies between specimens, but some of them match with our samples (Table 1). Numerous slide preparations (made by V. Koltun in the mid-20^th^ century) allow for the estimation of the diversity of spicule characters. The shape of spicules varied from stout, greatly spiny oxeas to thin, fusiform oxeas that bear minute spines.

### Genetic analysis

COI sequences of nine sponge samples of different colors and shapes were obtained. All sequences (MN431221–MN431229) were identical and have length 676 bp. A BLAST analysis revealed that the obtained sequences are most similar to the freshwater sponge *Ephydatia fluviatilis* (Linnaeus, 1759) (Spongillidae) and differ from its sequence by one nucleotide substitution. The obtained sequences were aligned with available GenBank sequences of Spongillida and *Vetulina stalactites* (the closest marine relative of Spongillida) and resulted in a 487 bp alignment, in which 28 characters were available for phylogenetic analyses. Phylogenetic reconstructions based on CO1 data obtained with BI and ML had similar topologies with poorly resolved phylogenetic relationships (tree not shown). Mean genetic distance between *M. tuberculata* and other freshwater sponges was 1% and between *M. tuberculata* and *V. stalactites* was 10%.

**Figure 4.**
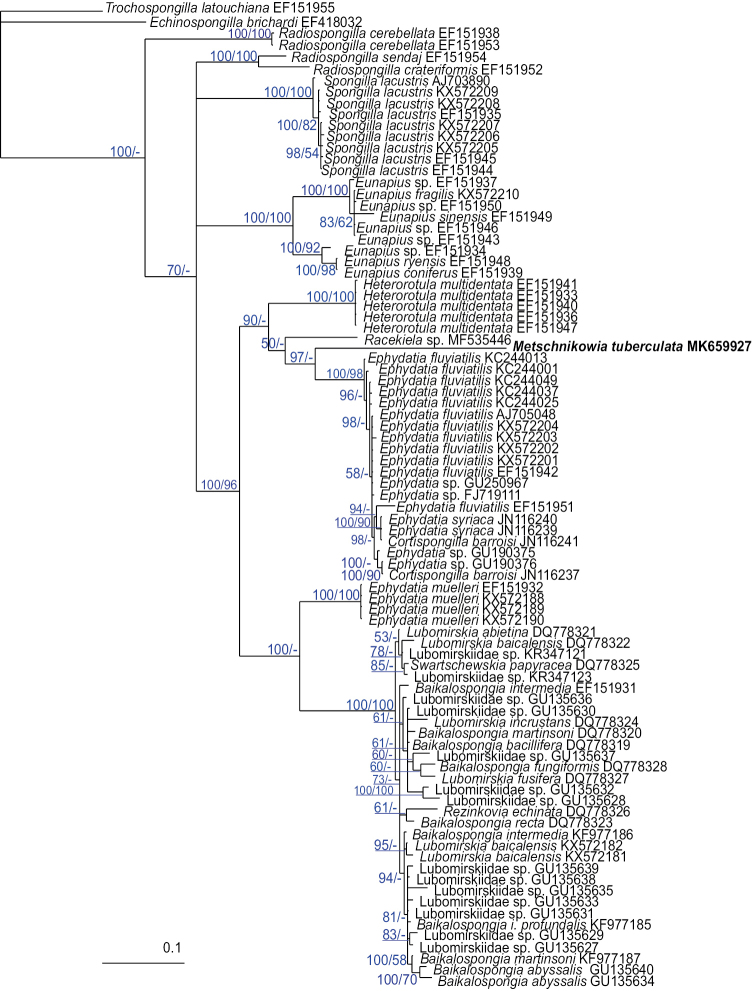
Bayesian phylogenetic tree based on comparisons of 873 bp of ITS 1 and ITS 2 sequences of Spongillida. Nodes are characterized by Bayesian posterior probabilities (%) followed by bootstrap percentages; a (–) indicates that a particular analysis supported the node at less than 50%, or supported an alternative phylogenetic arrangement in ML tree. *Trochospongilla latouchiana* (Spongillidae; GenBank EF151955) was used as the outgroup. Scale bar denotes substitutions per site.

ITS1 and ITS2 sequences were obtained from the same specimens. All sequences (MK659927–MK659935) were identical and have length 751 bp. A BLAST analysis revealed that the obtained sequences are most similar to the freshwater sponge *Ephydatia fluviatilis* (Linnaeus, 1759) (Spongillidae) and other Spongillida. The obtained sequences were aligned with available GenBank sequences of Spongillida and resulted in an 873 bp alignment, in which 437 characters were available for phylogenetic analyses. Marine sponges were not included in the analysis due to the high variability of ITS spacers making the alignment impossible. Phylogenetic reconstructions obtained with BI and ML had generally similar topologies, but the BI-tree shows higher support (Fig. 4).

The analyzed Caspian sponges form a clade with *Ephydatia syriaca* Topsent, 1910, *E. fluviatilis* and *Cortispongilla barroisi* (Topsent, 1892). This clade is included in the common clade with *Racekiela* sp. and *Heterorotula multidentata* (Weltner, 1895). All Lubomirskiidae form a strongly supported monophyletic clade with *Ephydatia muelleri* (Lieberkühn, 1856) as a sister species. Our results also support monophyly of the genus *Eunapius*. *Radiospongilla* is paraphyletic to all other species of Spongillidae.

## Discussion

During its existence, the Caspian Sea has repeatedly reconnected with the ocean. Now isolated, this waterbody retains typical marine features, such as characteristic water circulation, the structure of the water mass, hydrochemical properties, production of organic matter in the pelagic zone, geomorphological structure and distribution of organisms. On the other hand, the Caspian water is characterized by lower salt concentration (12–13‰ in the middle and southern parts) and modified salt composition (Karpinsky 2002). Having such a set of features, the Caspian became a cradle of specific fauna, partly of freshwater and partly of marine origin. Some Caspian invertebrates cannot be confidently referred to as animals of either origin, and sponges were among them. The Caspian sponges were known to have typical marine habitus (see pl. I, fig. 1–4 in Koltun 1968), but possess spicules similar to those of freshwater sponges. In addition, their distribution within the sea corresponded rather with the hypothesis of marine origin because sponges were found mainly in the Middle Caspian (Koltun 1962); these waters are much saltier than fresher North Caspian, where sponges were almost absent. Based on skeleton features, the Caspian sponges were considered as a freshwater monotypic family Metschnikowiidae (Manconi and Pronzato 2002). Based on genetic data, we have shown that the Caspian sponge *Metschnikowia tuberculata* indeed has a freshwater origin.

We observed two stable morphs (yellow globules and thin faded-green crusts) and many encrusting sponges with transitional colors. Grimm (1877) also described sponges “of all sorts of shades from pale yellow to bright red” forming “crusts up to 1.5 cm thick” or having various shapes, “sometimes as large as a child’s fist.” We find no distinctions between sequences of sponges with different appearances and insufficient dissimilarity of skeleton features. This allows us to consider the morphs as a single species, *Metschnikowia tuberculata*.

At the same time, we observed some tendency for an increasing number of oxeas’ spines in globular sponges compared with encrusting ones. Some encrusting sponges have spicules with minute spines, clearly seen under SEM, but not so obvious under a light microscope. Probably, it was this tendency that led Grimm to misidentify some sponges with smooth spicules as representatives of marine haplosclerid *Reniera* (accepted name *Haliclona*) (Grimm 1876). Dybowsky (1880) claimed that Grimm had missed the spines on the spicule surface. Afterward, Koltun (1962) figured out that sponges which had been identified by Grimm as *R. flava* have not only smooth spicules (common of haplosclerids), but also spiny. Moreover, sponges on Grimm’s drawing of *R. flava* (Grimm 1876, pl. 3, fig. 1) are very similar to those encrusting sponges collected by us due to their distinctive, regularly distributed oscula. Thus, we suggest that Grimm dealt with encrusting *Metschnikowia tuberculata* having reduced numbers of spines, not with some other sponge.

The Caspian fauna is considered to be in the process of formation because of significant morphological variety of fishes and benthic animals (Ustarbekov 2001), abundance of closely related species with transitional forms, wide ecological niches of species and low specialization and competitiveness compared with saltwater species (in the Black and Azov Seas) (Karpinsky 2002). The first two features are also attributable to sponges of another ancient lake, the Baikal. Being relatively young (Schuster et al. 2018b), its endemic sponges (family Lubomirskiidae) exhibit a large number of transitional morphological forms between species and possess overlapping morphotraits (Itskovich et al. 2015, 2017). Thus, the morphological variety of studied *M. tuberculata* seems not to be surprising.

Comparison of our specimens with the museum collection leaves no doubt that they represent *M. tuberculata*. However, spicules of some sponges from the slide collection stand out from others due to their large size and salient spines. Although freshwater sponges (Spongillida) are known for some spicule variability (e.g., Poirrier 1974, 1976), at present we cannot reveal the limits of variability in *Metschnikowia*. To determine the true diversity of the Caspian sponges more investigations of specimens from different locations and depths are required.

### Phylogeny

The current study is based on too few specimens and we certainly cannot claim all the Caspian sponges belong to the one species. Nevertheless, our results revealed that sponges of different morphs have identical ITS sequences. Taking into account that ITS sequences have a good resolution at species and generic levels in Spongillida (Itskovich et al. 2017), we conclude that all the studied samples belong to the one species, *Metschnikowia tuberculata*. Genetic distances show that *M. tuberculata* exactly belong to Spongillida.

Our data support the monophyly of freshwater sponges previously predicted by morphological data (Manconi and Pronzato 2002) and confirmed by molecular data (Addis and Peterson 2005; Itskovich et al. 2006; Meixner et al. 2007). Once being descended from marine sponges, Spongillida colonized fresh waters, probably through the coastal brackish waters (Manconi et al. 2013). However, some species remain resistant to slight salinity. For example, *Spongilla alba* Carter, 1849 is apparently associated with brackish waterbodies (Poirrier et al. 1987; Masuda and Satoh 1990; Gugel 1996). Another instance of salinity tolerance occurs in the widespread sponge *Ephydatia fluviatilis*, which can survive in mineralized waters like the Baltic Sea (Karlsson et al. 2012) and Lake Issyk-Kul (Weltner 1911).

Thus, the clustering of *Metschnikowia* with *Ephydatia* seems not surprising. This clade also supports the hypothesis of the formation of endemic species from cosmopolitan founders (Meixner et al. 2007; Erpenbeck et al. 2011). The high variability of the ITS spacers makes it difficult to align them unambiguously, which leads to low support for deeper nodes (Erpenbeck et al. 2019). Therefore, more markers are required for improving resolution of the trees.

CO1 of freshwater sponges, conversely, have low variability that resulted in an unresolved phylogeny within Spongillida both in our data and in previous analyses (Meixner et al. 2007; Erpenbeck et al. 2011; Itskovich et al. 2013). *Metschnikowia tuberculata* possesses new and distinct from *E. fluviatilis* CO1 haplotype. These data together with ITS data and morphological differences support the separate taxonomic status of the sponges.

Spongillidae is shown to be paraphyletic with respect to the malawispongiid *Cortispongilla barroisi*, agreeing with the results of Itskovich et al. (2013). Our results support the monophyly of Lubomirskiidae, the sponges of Lake Baikal. They form a strongly supported monophyletic clade with *Ephydatia muelleri* as a sister species, which is consistent with preceding analyses (Itskovich et al. 2008, 2015; Erpenbeck et al. 2019). However, *Radiospongilla* turns out to be paraphyletic. Except for this, the obtained results are in accordance with the previous data. They demonstrate the unresolved phylogeny of Spongillida at the family level (Erpenbeck et al. 2011, 2019; Carballo et al. 2018). We believe the revision of the taxonomy of Spongillida at the family level requires the addition of other molecular markers and genomic data.
